# Era of Synchronized Physiologic Leadless Pacing: A Novel Approach to Cardiac Pacing and Ongoing Development

**DOI:** 10.3390/jcm15031251

**Published:** 2026-02-04

**Authors:** Dhan Bahadur Shrestha, Jurgen Shtembari, Daniel H. Katz, James Storey, Ashlesha Chaudhary, Anuj Garg, Ajay Pillai

**Affiliations:** 1 Division of Cardiology, Department of Internal Medicine, Bassett Medical Center, 1 Atwell Rd, Cooperstown, NY 13326, USA; daniel.katz@bassett.org (D.H.K.); jaanstorey1@att.net (J.S.); 2 Division of Cardiology, Department of Internal Medicine, Carle Foundation Hospital, 611 W Park St, Urbana, IL 61801, USA; jurgen.shtembari@carle.com (J.S.); anuj.garg@carle.com (A.G.); 3 Department of Internal Medicine, Bassett Medical Center, 1 Atwell Rd, Cooperstown, NY 13326, USA; ashlesha.chaudhary@bassett.org; 4 Division of Cardiology, Virginia Commonwealth University, Pauley Heart Center, VCU Health System, Richmond, VA 23219, USA; ajay.pillai@vcuhealth.org

**Keywords:** leadless pacing, atrio-ventricular synchronous pacing, leadless cardiac resynchronization

## Abstract

Cardiac pacing has undergone a significant transformation in the last decade. Leadless pacing (LP), once only a conceptual idea stemming from the early interest in eliminating lead-related complications of transvenous pacemakers, has now become a reality in clinical practice. Since the introduction of the first human single-chamber asynchronous leadless ventricular pacing in 2012, atrioventricular-synchronized single- or dual-chamber leadless pacing systems have been approved for clinical use since 2020. Leadless cardiac resynchronization therapy (CRT) has shown optimistic results in case series and awaits its full utility in real-world clinical practice. With the successful feasibility study of leadless conduction system pacing, we are eagerly awaiting long-term safety and efficacy data on a large scale. Another important frontier is the development of self-rechargeable LP, which may be an ideal pacemaker for the future and may reduce the burden of multiple device replacements as batteries near the end-of-service. Totally extravascular percutaneous leadless pericardial micro-pacemaker system implantation is under development. In this state-of-the-art review, we examine the evolution of cardiac pacing, emphasizing the development and utility of LP to meet maximum physiological pacing needs, optimize atrioventricular synchrony and cardiac resynchronization, and broaden its indications.

## 1. Introduction

Since the first permanent pacemaker implantation in 1958, transvenous pacing (TVP) systems have undergone significant improvements, including smaller generators, longer battery life, improved lead quality, and expanded pacing modalities [1]. Despite these advances, approximately 10% of patients receiving transvenous pacemakers experience device-related complications, most commonly involving the pacemaker pocket or transvenous leads, such as infection, venous obstruction, lead failure, and challenges associated with lead extraction [1,2,3]. To overcome these limitations, Dr. Spickler tested the feasibility of a completely endocardial leadless pacemaker (LP) in a canine model in the 1970s [3]. This concept was translated into clinical practice in 2012 with the first successful human implantation of a self-contained leadless intracardiac pacemaker during the LEADLESS trial [4].

Two LP systems are currently approved by the United States Food and Drug Administration (US-FDA) and are in clinical practice. This includes the Micra™ Transcatheter Pacing System (Micra™ TPS, Medtronic, Minneapolis, MN, USA) and the Aveir™ pacemaker (Abbott Laboratories, Chicago, IL, USA). As completely intracardiac devices, LPs eliminate the need for device pocket creation and transvenous lead insertion, thereby avoiding pocket- and lead-related complications. Studies comparing LP and TVP have demonstrated comparable or lower complications rate with LP [2].

With the growing use of LP across diverse patient populations, including young patients, the limitations of single-chamber leadless pacing have become more apparent. This has driven the development of atrioventricular (AV) synchronized and physiologic pacing systems to minimize pacing-associated complications, such as pacemaker syndrome (PMS) and pacing-induced cardiomyopathy (PICM). Newer iterations of LP focus on improving overall cardiac AV synchrony, enabling physiologic pacing through engagement of the cardiac conduction system, and complete leadless cardiac resynchronization therapy (CRT) in appropriate patient populations. This review summarizes current-generation LP systems, approaches to cardiac AV synchrony, and emerging developments to further enhance physiologic pacing.

## 2. History of Cardiac Pacing

### 2.1. History of Traditional Transvenous Pacing

**Figure 1:**
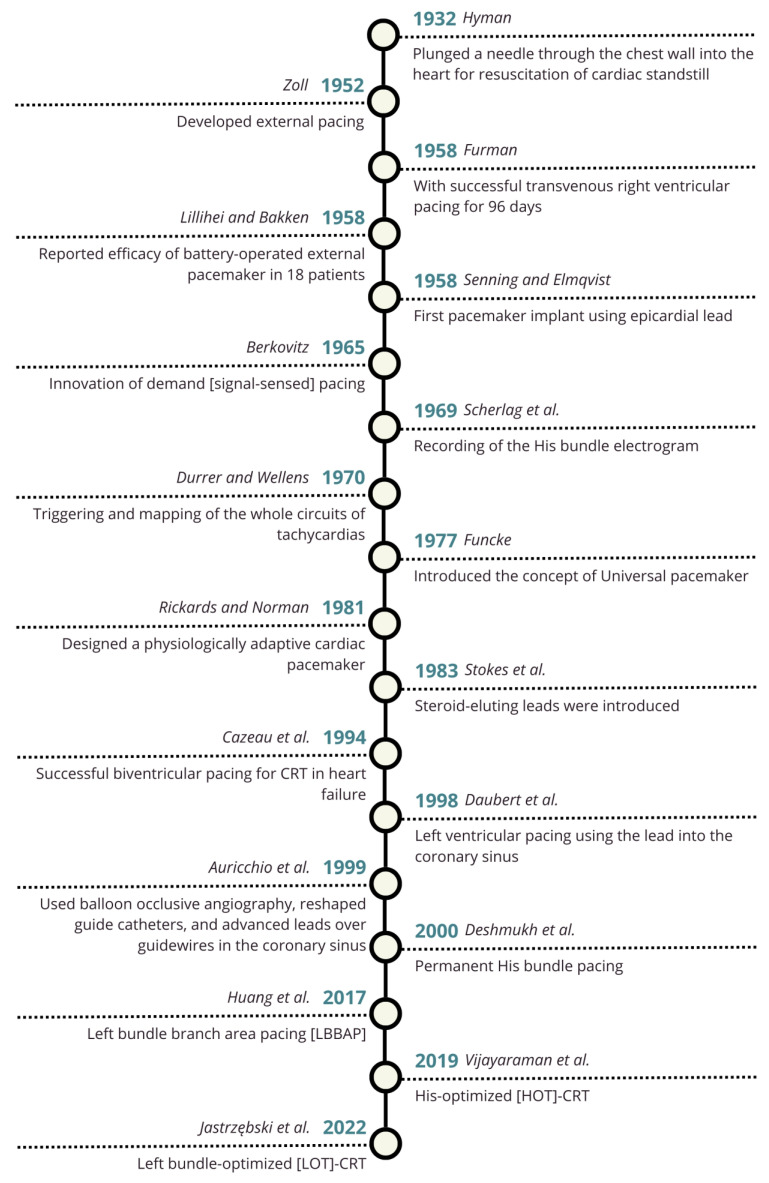
Timeline of pacing evolution [1,5,6,7,9,10,11,12,13,14,15,16,17,18]. Abbreviations: CRT: Cardiac resynchronization therapy; LBBAP: Left bundle branch area pacing; HOT-CRT: His-Purkinje conduction system pacing optimized CRT; LOT-CRT: Left bundle branch optimized CRT.

The first attempt at cardiac pacing was performed in 1932 by Alfred Hyman using a transthoracic needle approach to resuscitate a cardiac arrest [5]. Two decades later, in 1952, Paul Zoll introduced external cardiac pacing [6]. A major advancement followed in 1958, when Furman achieved 96 days of successful transvenous right ventricular (RV) pacing. In the same year, Lillehei and Bakken successfully used a battery-operated external pacemaker in 18 patients [1,7], and Senning and Elmqvist performed the first pacemaker implant using an epicardial lead. In subsequent years, the field of cardiac pacing evolved substantially, with improvements in lead design, advances in electrical mapping of the heart conduction system and arrhythmias, development of different pacemaker modes, and the introduction of CRT using the coronary sinus for left ventricular pacing (Figure 1) [1].

The efficacy of CRT has been evaluated in numerous trials across diverse populations, with the aim of expanding its role in heart failure management. The current Heart Rhythm Society guideline recommends CRT as a Class I indication for individuals with reduced ejection fraction (<35%), left bundle branch block (LBBB) with a QRS duration of ≥150 ms, New York Heart Association (NYHA) class II-IV symptoms, and guideline-directed medical therapy (GDMT). Additional Class II indications further broaden eligibility for CRT [8].

### 2.2. History of Leadless Pacing

Although transvenous permanent pacing systems evolved over time, they continue to carry a significant risk of complications and associated morbidity and mortality. Approximately 10% of patients undergoing TVP experience short-term complications related to the pulse generator, including pocket hematoma, infection, skin breakdown, or lead-related complications, such as lead dislodgement, pneumothorax, cardiac tamponade, or access-related complications [19]. Because the lead remains the most vulnerable component of the pacing system, the concept of a fully intracardiac LP emerged as a potential solution to these limitations and was subsequently elevated in clinical trials [3,4].

The first demonstration of leadless intracardiac pacing was reported by Spickler et al. in 1970 using a canine model [1]. More than four decades later, this concept was translated into clinical practice when the first leadless pacemaker was implanted in humans in 2012 [3,4]. The first LP, Nanostim™ (Nanostim Inc., Sunnyvale, CA, later acquired by St. Jude Medical and subsequently by Abbott), was a single-chamber ventricular LP with a screw-in-helix fixation mechanism. The LEADLESS trial was a multicenter, single-arm, prospective study evaluating the safety and efficacy of this device over three months and demonstrated the feasibility in human. Between December 2012 and April 2013, 33 patients were implanted with Nanostim™ across three participating centers, with an implant success rate of 97% (32/33). One patient died due to ventricular perforation with subsequent stroke; otherwise, pacing performance was acceptable at three months and one year of follow-up [4,20].

Subsequent evaluation in the larger LEADLESS II trial confirmed the feasibility of leadless pacing but also highlighted important safety limitations. Conducted across 56 centers in three countries, LEADLESS II demonstrated a successful implantation rate of 95.8% (504/526), with the primary efficacy endpoint met in 90% of the prespecified cohort (270/300). At six months, device-related serious events occurred in 6.7% (20/300) of patients in the primary cohort, including elevated pacing thresholds, device dislodgement, cardiac perforation, and vascular complications [21]. In October 2016, St. Jude Medical halted implantations of Nanostim™ LP due to reports of battery malfunction resulting in loss of telemetry and pacing output.

In 2015, Ritter et al. reported early experience with implantation of the tine-fixation Micra™ Transcatheter Leadless Pacing System (TPS, Model MC1VR01, Medtronic, Mounds View, MN, USA) [22]. The first Micra™ VR LP was implanted in 2013 in Austria. Micra™ VR is a single-chamber ventricular pacemaker, similar to Nanostim™, capable of pacing and sensing the right ventricle. Micra™ is smaller than Nanostim™, and allows the theoretical possibility of implanting up to three devices in the right ventricle without removing the prior LP. In 6 April 2016, the Micra™ Transcatheter Pacing System received US-FDA approval as the first leadless pacemaker, based on the results of the Micra™ Investigational Device Exemption (MICRA-IDE) study [23,24]. In the MICRA-IDE study, the device was successfully implanted in 99.2% (719/725) of patients. The primary safety and efficacy endpoints were met in 96% of the trial population, with low and stable pacing thresholds observed at six months in 98.3% of patients [24]. In 91% of the patient’s, pacing output was <1.5 V at a 0.24 ms pulse width, with an estimated projected battery longevity of 12.5 years, and 94% of devices projected to last longer than 10 years [24].

Despite these favorable outcomes, first-generation leadless pacemakers were limited by their inability to provide AV synchrony, as they were restricted to VVI pacing modes. This limitation narrowed their applicability to selected patient populations and prompted the development of next-generation systems capable of AV-synchronous pacing. Medtronic subsequently introduced the Micra™ AV synchronous pacing system, which received the US-FDA approval in 2020 following the MARVEL-1 (Micra™ Atrial tRacking using a Ventricular accelerometer) and MARVEL-2 studies [25,26]. The Micra™ AV leadless pacing system was designed to provide ventricular pacing with AV synchrony (VDD). The timeline of LP evolution is summarized in Table 1, and a comparison of available LP systems is provided in Table 2.

## 3. Reported Complications with LP

Overall complication rates with LPs are lower than those observed with TVP, largely because LPs eliminate device pockets and transvenous leads. However, studies have shown that LPs are associated with higher rates of pericardial effusion, cardiac perforation, and vascular complications [2]. In major leadless pacing system trials and registries, short-term complication rates ranged from 2% to 10%, and long-term complications were reported to be less than 5% (Table 3 and Table S1). The majority of reported complications include procedure-related cardiac perforation, vascular injury or bleeding, and arrhythmias. In addition, although LPs are increasingly accepted in clinical practice, end-of-service (EOS) management remains a challenge due to finite battery life, as removal of chronically implanted LPs can be difficult because of endocardial encapsulation. As a result, only a limited number of LPs can be safely implanted over an individual’s lifetime while abandoning the prior devices in situ [30].

### 3.1. Common Complications with LP

Common complications associated with LP implantation include myocardial injury or perforation and pericardial effusion or tamponade, as well as vascular access site complications such as pseudoaneurysm, fistula, bleeding, and hematoma. Although the absolute incidence of these events is low, rates of cardiac perforation and vascular complications are higher with LP than with TVP in major clinical trials and post-market registries [2]. The risk of myocardial perforation and pericardial effusion may be higher with multiple device repositioning attempts. Vascular complications are primarily related to the use of large-bore femoral venous sheaths, and the routine use of ultrasound-guided vascular access has been shown to reduce this risk.

Device dislodgement during implantation is an infrequent but recognized adverse event with LPs. In major LP trials and registries, device dislodgement has been reported in less than 0.5%. This rate is higher with atrial LP due to anatomical constraints [31]. In the AVEIR DR i2i study, intraprocedural dislodgment occurred in 2% of cases, predominantly involving atrial LPs due to inadequate helix fixation, with an additional 2% experiencing postprocedural dislodgement limited to atrial devices [32]. Most dislodged devices were successfully retrieved, followed by implantation of another LP or an alternate pacing system. Procedural complication rates have continued to improve with increasing operator experience and refinements in device design, including the novel double-screw-in-helix used in atrial LPs [31,32].

Tricuspid regurgitation (TR) is a well-recognized complication of TVP, often resulting from mechanical interaction between transvenous leads and the tricuspid valve apparatus. In the absence of transvenous leads, LPs were initially expected to reduce the risk of TR. However, with higher septal LP implantation to potentially engage the conduction system and reduce PICM, worsening TR has been observed in up to 43% of patients receiving LPs [33]. The distance between the tricuspid valve annulus and the LP has been identified as an independent predictor of TR progression, with each 1-mm decrease associated with an approximately 15% increase in the risk of significant TR. Other clinical and echocardiographic parameters, including baseline TR, RV pacing burden, mitral regurgitation, and ventricular strain parameters, were not significantly associated with TR progression. These findings suggest that worsening TR may result from direct mechanical interference of the LP with the tricuspid apparatus [33]. Careful preprocedural planning and device positioning may therefore reduce the risk of both TR progression and PICM following LP implantation [34].

With long-term RV pacing using VVI mode, cases of PICM have been reported in patients with LPs. Although PICM occurs in a minority of LP recipients, its underlying mechanisms remain poorly understood [35]. PICM is defined as a new reduction in left ventricular ejection fraction (LVEF) to <50% without an alternative cause or a ≥10% decrement in LVEF from baseline. High RV pacing burden (≥40%) is the principal risk factor, with additional associations reported for male sex, older age, lower baseline LVEF, and wider paced QRS duration. In an observational study with approximately 1.5 years of follow-up, PICM occurred in 7.8% of LP recipients and was most frequent in those with apical or apical septal LP placement. In contrast, the lowest rates were observed with mid- to high-septal (HS) implantation [36,37].

Comparative analyses have further demonstrated that in patients with non-atrial fibrillation bradycardia, asynchronous VVI pacing with a ventricular LP is associated with higher rates of heart failure hospitalization and worsening TR compared with synchronous DDD-TVP. This difference is likely related to electromechanical desynchrony associated with asynchronous VVI pacing [38]. To mitigate these effects, lower programmed pacing rates may be used to promote intrinsic conduction when feasible. In patients without permanent atrial fibrillation, AV-synchronous leadless pacing using Micra™ AV or Micra™ AV2 is generally preferred over asynchronous ventricular pacing. With the advent of dual-chamber leadless systems such as Aveir™ DR, most pacing modalities can now be achieved, allowing improved AV synchrony and potentially reducing pacemaker syndrome and PICM. Placement of LPs in the mid- to high-septal region may further reduce the risk of PICM through engagement of the conduction system. However, this hypothesis requires further confirmation in larger prospective or randomized studies. Leadless left bundle branch area pacing currently represents a promising next step in the evolution of physiologic leadless pacing.

**Table 3:** Main Leadless Trials complications summary.

Landmark Studies	LP Model	Follow-Up Duration (mo)	Short-Term Complications (%)	Pericardial Effusion/Perforation (%)	Vascular Complications (%)	Long-Term Complications (%)	Infection (%)
**LEADLESS study** [4,20]	Nanostim™	38	6.1	3.0	0.0	3.0	0.0
**LEADLESS-II** [21]	Nanostim™	6	5.8	1.5	1.1	0.6	0.0
**MICRA-IDE** [24]	Micra™ VR	16.4	2.9	1.4	0.7	1.1	0.0
**MICRA-CED** [39]	Micra™ VR	22.2	8.4	0.8	1.4	5.0	<0.2
**MICRA-PAR** [40]	Micra™ VR	51.1	2.5	0.4	0.6	1.8	0.1
**MICRA AV-CED** [41]	Micra™ AV	6	9.1	1.4	1.0	3.6	<0.2
**LEADLESS-II phase II** [42]	Aveir™ VR	14.4	2.9	1.9	1.0	1.9	0.0
**Aveir DR i2i Study** [27]	Aveir™ DR	3	9.7	0.7	`0.7	-	0.0

### 3.2. Rare Complications Reported with LP

Capture threshold at implantation is essential, as it predicts long-term outcomes, particularly battery longevity. An initial capture threshold of ≤1.0 V at 0.24 ms is considered appropriate for LP implantation. Acute increases in pacing threshold and impedance after successful LP implantation have been reported in case reports and may be related to myocardial injury at the time of LP implantation or to an acute thrombus between the LP and the myocardium. Close follow-up with device interrogation is reasonable, as thresholds and impedance may normalize over several weeks if there is no apparent cause for the threshold rise, such as a broken fixation tine [43,44]. Rate-dependent increases in capture threshold during bradycardia have also been reported and are proposed to result from inflammation-induced conduction block or microdislodgement. Threshold testing at different pacing rates during LP implantation may help ensure stable device performance [45]. Intermittent loss of telemetry data has also been reported and is thought to be due to body habitus or deep breathing; this typically resolves with patient repositioning, improving telemetry communication [46].

Rare conduction disturbances have been reported following septal LP implantation aimed at engaging the conduction system. Specifically, implantation of the Micra™ VR device in the high-to-mid interventricular septum has been associated with transient new-onset 2:1 atrioventricular block in isolated cases [47]. Careful patient selection is important, as patients with underlying myocardial disease, such as occult coronary disease or myocardial scarring, may develop arrhythmias, including ventricular tachycardias (VT). These arrhythmias may be triggered by device implantation or pacing-related premature ventricular complexes and re-entrant circuits in the presence of an underlying substrate [48].

Infectious complications involving LPs are uncommon. Compared with transvenous pacing systems, LPs demonstrate relative resistance to bacterial colonization owing to their smaller surface area and progressive encapsulation by endocardial tissue. Consequently, LPs are often favored in patients at elevated risk for device-related infection or infective endocarditis. In the Micra™ IDE study, serious infectious events occurred in 2.2% of patients, and were predominantly transient bacteremia with only three cases of endocarditis [49]. Nevertheless, cases of fungal LP endocarditis and late-onset bacterial endocarditis have been reported in immunocompromised patients or in those with factors that prevent complete device reendothelialization [50,51].

## 4. Current Generation Leadless Pacing and AV-Synchronization

### 4.1. Single-Chamber Leadless Pacing

The Nanostim™, the first single-chamber ventricular leadless pacemaker studied in a human trial, was withdrawn from the market in 2016 due to battery-related safety concerns. Subsequently, Micra™ VR received Conformité Européenne (CE) Mark in 2015 and US-FDA approval in 2016, and was the only LP available during that period. The second-generation LP Micra™ AV received US-FDA and CE marks in 2020. Micra™ AV was designed to overcome a limitation of Micra™ VR by enabling AV synchronization, defined as a P-wave followed by a QRS complex within 300 ms, using a three-axis accelerometer algorithm that allows sensing of atrial mechanical activity [25,26].

**Figure 2:**
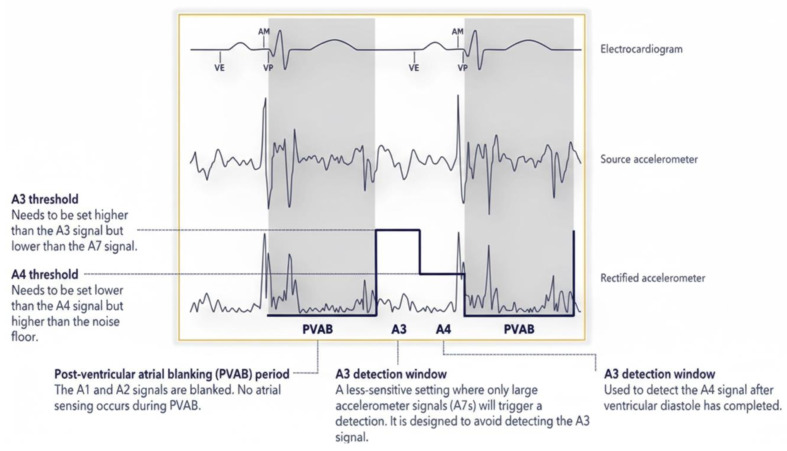
Micra™ AV2 accelerometers signals. Image provided courtesy of Medtronic. ©2025 Medtronic. All rights reserved. Used with the permission of Medtronic.

Micra™ AV achieves AV synchrony by detecting atrial mechanical activity using a three-axis accelerometer, allowing ventricular pacing to follow atrial contraction within a predefined timing window. Data from the Micra™ Accelerometer Sensor Sub-Study (MASS) and MASS2 studies identified four distinct accelerometer signals corresponding to specific cardiac activities. As depicted in Figure 2, A1 corresponds to isovolumetric contraction and closure of the atrioventricular valves (mitral and tricuspid), A2 corresponds to closure of the pulmonic and aortic valves, A3 corresponds to passive ventricular filling, and A4 corresponds to atrial contraction. On Doppler echocardiography, the mitral inflow E-wave correlates with A3, whereas the A-wave corresponds with the A4 signal. On the electrocardiogram, AM marking indicates atrial mechanical contraction (A4), and VE indicates the end of the A1-A3 ventricular signal (Figure 2) [25,52]. AV synchrony broadens the utility of Micra™ AV in patients with sinus node disease. In the MARVEL-2 trial, Micra™ AV demonstrated AV synchrony of 89.2% at rest, which ranged from 29% to 75% in an ambulatory setting, and improved to 40–86% after multiple follow-ups and device reprogramming [53,54]. However, when heart rate is elevated (>115 bpm) or diastolic dysfunction (E/A > 1.5) is present, sensing of atrial contraction is difficult, likely due to fusion of A3 and A4 signals with motion-related signals.

To achieve optimal AV synchrony, it is recommended to disable the Auto A3 threshold and tracking check in all patients. Individuals with high-grade AV block should have the AV conduction mode turned off. Following automatic Micra™ AV auto-setup, the manual atrial mechanical (MAM) test, along with assessment of patient profile and activity, should be performed to improve AV synchrony. Micra™ AV may be appropriate for individuals who do not require AV synchrony at higher heart rates. Disabling the A3 signal or setting the A3 threshold higher than the A3 signal amplitude may help prevent undersensing during sustained high heart rate (>85 beats per minute) [52]. Detailed programming recommendations are beyond the scope of this manuscript; and readers are referred to the manufacturer’s guide and relevant societal guidelines [52].

To address challenges related to AV synchrony with tachycardia and battery longevity, the next-generation Micra™ AV2 and Micra™ VR2 devices received US-FDA approval in 2023 and European Union CE Mark approval in 2024 [55]. Micra™ AV2 offers a higher upper tracking rate up to 135 beats per minute. The latest generation of Micra™ devices (Micra™ AV2 and Micra™ VR2) also provides improved battery longevity and enhanced programming features. Collectively, these refinements represent a meaningful advancement in single-chamber leadless pacing technology. A summary of outcomes with AV-synchronous single- and dual-chamber leadless pacing systems is provided in Table 4.

The successor to Nanostim™, Aveir™ VR, incorporates modifications to the battery and docking button and received US-FDA approval on 31 March 2022, following the results of the LEADLESS II Phase 2 study, with CE Mark approval granted in 2023 [42]. Similarly to Nanostim™, Aveir™ VR uses a screw-in helix fixation system and is slightly thinner and longer than Micra™ VR. Aveir™ VR relies on a temperature sensor for rate-responsive pacing, while Micra™ VR uses a three-axis accelerometer. Both Aveir™ VR and Micra™ VR provide right ventricular pacing and sensing in VVI(R) mode. A comparison of currently available LPs is provided in Table 1.

**A.**  **Leadless left bundle branch area pacing**

Traditional RV pacing causes electromechanical desynchrony of the heart, which over time can lead to pacing-induced cardiomyopathy and heart failure. To minimize this complication, physiological pacing strategies, particularly His-Purkinje bundle pacing (HPBP), has been extensively studied over the last three decades. However, His bundle pacing is technically challenging due to anatomic complexity, a steep learning curve, and a higher pacing threshold, prompting the search for alternative pacing techniques [56]. In 2017, Huang et al. reported pacing strategies engaging the left bundle branch, which subsequently received US-FDA approval in 2022 and have been since widely adopted [16]. At present, left bundle branch area pacing (LBBAP) is considered a physiological pacing strategy, with studies demonstrating its superiority over biventricular pacing for cardiac resynchronization in heart failure patients [56,57].

Conduction system pacing (CSP) has also been explored using the Micra™ VR leadless pacing system, with implantation in the high-to-mid interventricular septum. Septal implantation of LPs aims to engage the conduction system and minimize the PICM associated with right ventricular apical pacing [37]. However, septal implantation has been associated with more tricuspid valve regurgitation [33]. Recently, Reddy et al. reported the first human LP system that engages LBBAP. In this feasibility study, 14 patients were enrolled; LP was successfully implanted in 10 patients. Among these, 5 patients achieved LBBAP, 3 had deep-septal pacing, and 2 had RV-septal pacing. Two serious adverse events occurred within one month, including worsening of tricuspid regurgitation in one patient, and significant hematoma formation at the internal jugular vein access site in another; both were managed conservatively [28]. Following this proof-of-concept feasibility study, further investigation with longer-term follow-up is warranted. According to Abbott, the Aveir™ CSP has received US-FDA Breakthrough Device Designation, and additional studies are awaited to evaluate its safety and effectiveness [58].

**B.**  **Leadless Bachmann bundle pacing**

Since Thomas N. James described internodal pathways from the sinus node to the AV node in 1963, there have been significant efforts to study atrial electrophysiological properties and pacing involving Bachmann’s bundle (BB) [59]. BB was defined as secondary branches of the superior vena cava arising from the anterior pathway, coursing from the sinus node anteriorly toward the left atrium. Disruption of these internodal pathways has been hypothesized to contribute to the development of several atrial tachyarrhythmias. BB serves as the preferential pathway for interatrial conduction, and delay, or diseases affecting the BB leads to characteristic changes in P-wave morphology and prolongation of interatrial conduction time. Individuals with inter-atrial conduction block and atrial fibrosis have a substantially higher incidence of atrial fibrillation or atrial flutter compared with the control population of similar age, sex, clinical characteristics, and diagnosis of heart failure. Non-physiologic right atrial pacing that does not engage the BB has been shown to increase the incidence of atrial fibrillation. In contrast, preferential pacing via the BB has been associated with a reduction in atrial fibrillation burden [59].

Recently, Hollis et al. reported the first successful case of leadless Bachmann’s bundle area pacing (BBAP) for atrial resynchronization in a patient with marked atrial desynchrony, sinus node dysfunction, prolonged and superiorly directed P wave, and a history of atrial fibrillation in the setting of prior multiple cardiac surgeries [29]. Following BBAP, a significant reduction in conduction time from the right atrium to the lateral left atrium was observed, along with shortening of the paced P wave duration [29]. Further studies are needed to evaluate the safety, durability, and long-term clinical impact of leadless BBAP, including its potential role in reducing atrial fibrillation burden and recurrence.

### 4.2. Dual-Chamber Leadless Pacing

Recently, Abbott Medical evaluated the first dual-chamber LP with improved helix-fixation, Aveir™ DR. This system supports all pacing modes, uses implant-to-implant (i2i) communication to achieve AV synchrony with dual-chamber pacing, and incorporates a temperature-based sensor for rate modulation. Safe implantation of an atrial LP in a thin atrial wall and maintenance of reliable beat-to-beat communication with acceptable battery longevity remain major challenges with Aveir™ DR. The atrial LP uses conductive communication through blood and tissue and incorporates a dual-helix fixation design to reduce the risk of device dislodgement and perforation. 100% AV synchrony was observed across different postures, with approximately 99% successful device-to-device communication in preclinical animal studies [60,61].

The Aveir™ DR i2i first-in-human trial evaluated dual-chamber LP in patients with sinus node dysfunction or AV block. Implantation was successful in 98.3% (295/300) of patients, with a 3-month complication rate of 9.7% and a perforation rate of 0.7%, comparable to TVP. However, atrial LP dislodgement was observed in 3.3% of patients at 3 months [27]. The base of the right atrial appendage has been identified as the optimal site for atrial LP implantation to facilitate device-to-device communication [62]. Adequate atrial pacing and sensing were observed in 90.2% and 92.8% of patients at 3 months and 1 year, respectively, with the overall electrical performance comparable to TVP. AV synchrony ≥ 70% was achieved in 97.3% of patients, and was maintained across different postures ≥ 96.5% of the time. Atrial-to-ventricular and ventricular-to-atrial communication were successful in 90.3% and 87.5% of beats, respectively, and i2i communication appeared to preserve the battery longevity [27].

**Figure 3:**
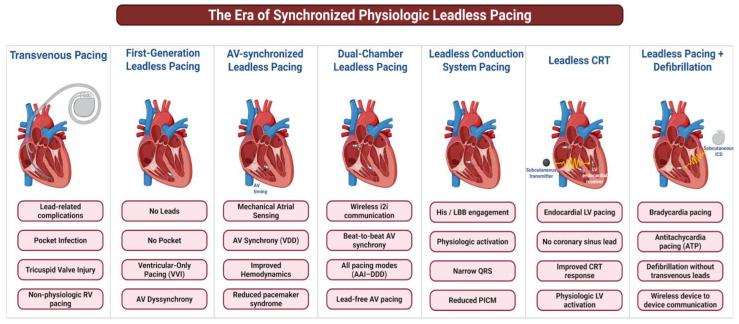
Central illustration showing different leadless pacing options in the era of synchronized physiologic leadless pacing, from initial dual chamber transvenous pacing to completely leadless/extravascular CRT/ICD. Created in BioRender. Shrestha et al. (2026) https://BioRender.com/tgko66h (accessed on 30 January 2026).

Based on these findings, Aveir™ DR received US-FDA approval in 2023 and CE Mark approval in 2024. Importantly, the system allows modular implantation, enabling initial use as a single-chamber atrial or ventricular LP with subsequent upgrade to dual-chamber pacing if clinically indicated, thereby reducing procedural risk, cost, and battery burden [63]. Compared with Micra™ AV, Aveir™ DR provides superior AV synchrony, which may reduce the risk of PMS and PICM [63]. Careful consideration of the ventricular device on the mid-to-high-septal region further improves i2i communication and AV synchrony, minimizing so desynchrony and associated complications. Central illustration (Figure 3) shows the currently available and feasible leadless pacing options for synchronized physiologic leadless pacing in real-world practice and clinical studies, contrasted with transvenous pacing.

### 4.3. Comparison of Aveir™ and Micra™

As shown in Table 1, comparison of commercially available LPs demonstrates that the second-generation Micra™ VR2 and Micra™ AV2 are successors to their first-generation devices, offering improved programming features and longer battery life. Micra™ VR2 is a single-chamber ventricular LP without atrial sensing, whereas Micra™ AV2 provides atrial-sensing using a three-axis accelerometer to enable AV-synchrony. The current generation of Aveir™ AR and Avier-VR devices can be implanted as single-chamber atrial and ventricular LPs, respectively, and can be implanted as a dual-chamber LP system (Aveir™ DR) when clinically appropriate [63]. Aveir™ DR uses conductive i2i communication between atrial and ventricular devices, achieving near-complete communication and enabling the implementation of essentially all pacing modes. This contrasts with Micra™ AV2, in which optimal AV synchrony may be limited at higher heart rates or during physical activity due to motion-related noise and shortened diastole, leading to fusion of A3 and A4 signals and reduced atrial sensing accuracy [52,60].

A key practical distinction between the two platforms relates to fixation and delivery systems. Micra™ devices use passive fixation with four nitinol tines and are delivered via a femoral venous approach with a dedicated delivery catheter. Micra™ devices do not have a dedicated mechanism for routine retrieval, and removal, when required, is typically performed using snare-based techniques [64]. In contrast, Aveir™ devices use an active screw-in helix fixation and are designed with features that facilitate engagement by a dedicated retrieval catheter, supporting planned device retrieval and replacement [65]. Rate-responsive pacing is achieved using different sensor technologies: Micra™ devices rely on a three-axis accelerometer, whereas Aveir™ devices use a temperature-based sensor. Aveir™ devices are longer and thinner and are associated with longer battery life than the Micra™ device. Micra™ uses a nitinol tine with passive fixation, while Aveir™ uses an active screw-in helix system. Both platforms are MRI conditional. Micra™ supports remote monitoring via Carelink, but this feature is currently unavailable for Aveir™ devices. For EOS management, Aveir™ has a dedicated explant system, whereas Micra™ does not.

Despite these technical and functional differences, comparative analyses demonstrate similar short-term complication rates and overall pacing effectiveness between Micra™ and Aveir™ systems [66]. Taken together, Aveir™ DR offers the flexibility of initial single-chamber implantation with the option to upgrade later to dual-chamber pacing, longer battery longevity, and near-complete AV synchrony with access to all pacing modes, making it a highly versatile LP platform at present. Aveir™ VR may be particularly well-suited for active patients who require AV synchrony at faster heart rates.

## 5. Cardiac Resynchronization with Leadless Pacing System

Approximately 30–40% patients with heart failure receiving conventional CRT either fail to respond or are unable to receive the CRT because of unfavorable coronary sinus anatomy. To overcome these limitations, alternative approaches to engage the conduction system, such as LBBAP-CRT, are under active investigation, with published data favoring LBBAP-CRT [56].

### 5.1. WISE-CRT Modular Pacing

**Figure 4:**
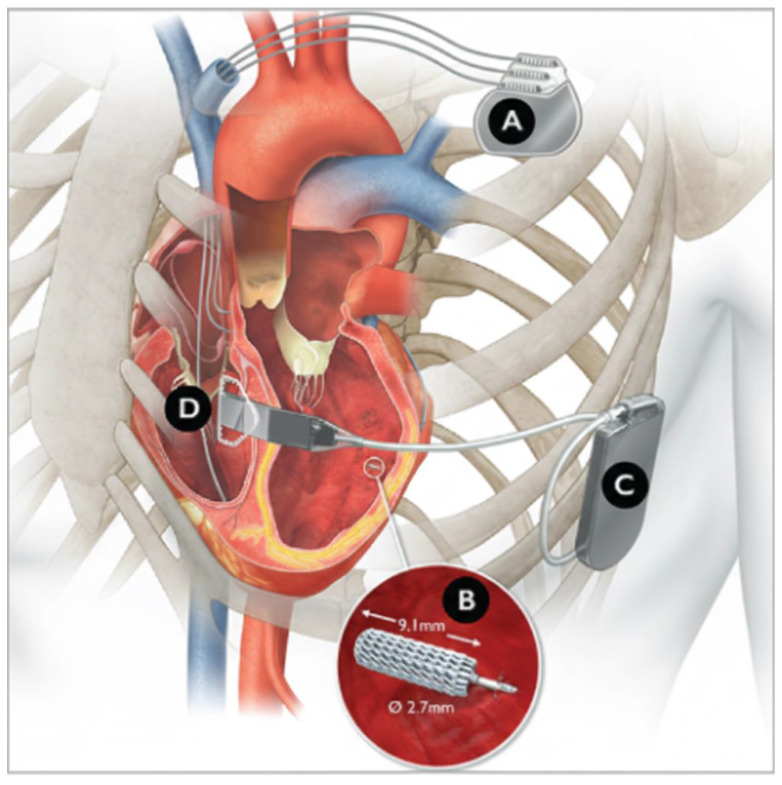
WISE-CRT system with its components. “Existing co-implant providing right ventricular pacing (**A**); the receiver-electrode converts ultrasound into electrical energy to pace the LV from the endocardium (**B**); the battery powers the transmitter; implanted subcutaneously at the left midaxillary line (**C**); the phase array transmitter that is synchronized with right ventricular pacing pulse and transmits ultrasound energy to the receiver-electrode (**D**).” Adapted from [71] Singh JP et al., abiding by open access article distribution under the terms of the CC-BY-NC-ND license.

The Wireless Stimulation Endocardially for Cardiac Resynchronization (WiSE-CRT) system (EBR Systems, Sunnyvale, CA, USA) is a hybrid pacing platform that delivers ultrasound energy to an LV endocardial receiver electrode, synchronized with RV pacing provided by a co-implanted pacemaker or defibrillator system (dual chamber pacemaker, dual chamber defibrillator, or CRT-pacemaker/defibrillator), Figure 4. The system consists of a subcutaneously implanted pulse generator with an ultrasound transmitter and an endocardially implanted receiver electrode. Endocardial LV stimulation facilitates physiological LV myocardial depolarization that more closely resembles intrinsic endocardial-to-epicardial activation [67,68]. This approach overcomes limitations related to coronary venous anatomy and avoids the need for long-term anticoagulation with an LV endocardial-implanted pacemaker lead, as in the older-generation CRT system with conventional transvenous leads [67,68].

The WiSE-CRT system was developed based on observations from the ALSYNC (ALternate Site Cardiac ResYNChronization) study, which demonstrated the benefit of LV endocardial pacing using a transvenous lead in patients with heart failure who were non-responders to epicardial coronary sinus pacing [69]. High procedural success rate and favorable clinical responses were subsequently reported in early studies, including the SELECT-LV and the SOLVE-CRT roll-in phase I study, in which WiSE-CRT systems were implanted [68,70]. Continued evaluation of the SOLVE-CRT trial, a multicenter prospective study, further validated these, demonstrating that the WiSE-CRT system, a novel leadless LV pacing system, is safe and effective in patients requiring CRT upgrade when coronary sinus lead placement is not feasible or when a prior CRT system has failed [71].

The WiSE-CRT received European CE Mark approval in 2015 following results of the initial feasibility study [67] and the SELECT-LV [70] study. On 15 April 2025, it also received US-FDA approval based on the results of the SOLVE-CRT trial [71]. A summary of clinical outcomes with the WiSE-CRT system is provided in Table 5.

### 5.2. Complete Leadless Cardiac Resynchronization

Completely leadless cardiac resynchronization therapy using the Micra™ leadless pacemaker in combination with the WiSE-CRT system has been reported [73,74]. In a case series from Europe, patients received Micra™ LP because implantation or upgrade to conventional CRT was not feasible due to infection or anatomical limitations. The combined Micra™ WiSE-CRT approach was associated with significant QRS narrowing and substantial improvement in LV function, including reverse remodeling and symptomatic improvement at 6 months follow-up [75]. Larger randomized studies are required to establish the long-term safety and efficacy of this fully leadless CRT strategy before widespread clinical adoption.

## 6. Leadless Pacemaker with ICD

### 6.1. LP with S-ICD

The subcutaneous implantable cardiac defibrillator (S-ICD) has inherent limitations, as it does not provide bradycardia pacing or anti-tachycardia pacing (ATP). To address these limitations, S-ICD co-implantation with commercially available LPs has been reported to provide bradycardia pacing and ATP therapies from LP in selected patient populations [76,77]. This combined approach avoids transvenous lead-related complications associated with conventional transvenous pacing and defibrillation systems. In preclinical studies, ATP-enabled LPs combined with S-ICD systems showed acute and chronic safety and performance, with successful wireless communication between devices [78]. More recently, a modular pacing-defibrillator system incorporating an LP capable of wireless communication with an S-ICD was evaluated in the multinational MODULAR-ATP study (Effectiveness of the EMPOWER™ Modular Pacing System and EMBLEM™ Subcutaneous ICD to Communicate Anti-tachycardia Pacing) [77]. In this study, 97.5% of patients were free from major LP-related complications, and 98.8% achieved successful wireless device-to-device communication. ATP successfully terminated 61.3% of ventricular arrhythmia episodes, with no reported communication failure [77]. The Modular-ATP system is currently awaiting US-FDA and CE approvals for commercial use.

### 6.2. LP with EV-ICD

The Aurora extravascular implantable cardioverter-defibrillator (EV-ICD; Medtronic, Minneapolis, MN) received European CE Mark approval in April 2023 and US-FDA approval in October 2023 following results from the EV-ICD pivotal trial [79]. While the EV-ICD provides extravascular defibrillation and ATP capability, it does not offer chronic bradycardia pacing. To address this limitation, early feasibility studies have explored combining LPs with EV-ICD systems [80]. Further investigation is required to assess the safety and reliability of device interaction and the risk of inappropriate therapy for broader clinical application.

## 7. Comparison of LP vs. TVP

With increasing experience, complication rates associated with LPs continue to decline as implanters overcome the initial learning curve. Traditional transvenous right ventricular pacemaker leads are typically implanted at the RV apex and are associated with a higher risk of PICM cases, particularly with a high RV pacing burden. Ongoing improvements in device design and implantation techniques may further reduce LP-related complication rates in the future. During implantation, minimizing device repositioning is important, as repeated repositioning increases the risk of cardiac perforation [81].

Careful patient selection is pivotal when considering LP implantation, as patient comorbidities, individual risk factors, preferences, pacing dependency, and the potential need for biventricular pacing should be weighed when choosing between single-chamber or dual-chamber LP systems versus transvenous pacemakers [81]. LPs are most commonly implanted via right femoral vein access, although the internal jugular vein has been reported as an alternative approach. Ultrasound-guided vascular access has been shown to reduce complications at the access site. With increasing operator experience, the risks of device dislodgement and cardiac perforation continue to decrease.

Individuals at high risk for bleeding or infection, including those with end-stage renal disease on dialysis, diabetes, frailty, chronic liver disease such as cirrhosis, or immunocompromised states, may derive particular benefit from LPs compared with TVPs. However, LP technology remains in an early phase of adoption, and it is currently more costly than TVP. In this context, the European society of cardiology (ESC) guidelines recommend LP as an alternative to TVP in patients without upper-extremity venous access or with prior or high risk of pocket infection (Class IIA), and in patients with limited life expectancy following shared decision-making (Class IIb) [82].

## 8. Use of LP as a Bailout in Rare and Challenging Situations in Specific Populations

Leadless pacemakers have emerged as valuable bailout options in selected patient populations in whom conventional transvenous pacing is technically challenging or associated with unacceptably high risk. Their entirely intracardiac design allows pacing in clinical scenarios where venous access is limited, infection risk is high, or prior cardiac interventions preclude safe transvenous lead placement.

A. Leadless pacing in valvular heart disease following valve intervention

Multiple case series have reported successful LP implantation during or after various tricuspid valve surgeries, repairs, or replacements, demonstrating its feasibility in these settings [83]. Similarly, LPs have been successfully implanted during open heart mitral and tricuspid valve surgery and concurrently with, or following, transcatheter aortic valve replacement. These approaches provide effective pacing without interference with prosthetic valves or repaired structures [84,85,86].

B. Leadless pacing in patients with limited vascular access

LP implantation has been successfully performed in patients with limited or hostile venous access, including those with an inferior vena cava filter, unsuccessful femoral delivery due to a small right atrium, difficult angulation, or other anatomical constraints. In such cases, internal jugular venous access may be preferred, as it offers a shorter route to the right atrium, bypasses the tortuous femoral veins, facilitates easier manipulation of the delivery system, and may result in a less painful procedure for the patient [87].

C. Leadless pacing in patients undergoing radiation therapy

With the increasing use of combined chemotherapy, radiotherapy, and immunotherapy, management of patients with cardiac implantable electronic devices undergoing thoracic radiation presents unique challenges. Radiotherapy for breast cancer, lung cancer, or mediastinal lymphomas may expose conventional pacemakers or defibrillators to radiation doses that can cause device malfunction. In these situations, LPs such as Micra™ or Aveir™ may serve as a bridge, allowing delivery of necessary malignancy treatment while avoiding direct radiation exposure to a traditional transvenous pacemaker generator and leads [88]. Although radiation is unlikely to cause significant damage to leadless pacemakers, device interrogation before and after radiotherapy is recommended to identify any potential device or software dysfunction [88].

D. Leadless pacing after heart transplantation

Approximately 5% of patients require permanent pacing following heart transplant (HT), most commonly due to sinus node dysfunction or atrioventricular block related to surgical trauma, ischemia, cardiac denervation, or autonomic dysfunction [89]. Given the immunocompromised state of HT recipients and their elevated risk of infection, LPs are increasingly considered a safe and effective alternative to TVP. Case series have reported implantation of single-chamber ventricular LPs (Micra™ VR, Micra™ AV) as well as dual-chamber Aveir™ DR systems following HT [90,91]. While early outcomes are encouraging, larger comparative studies are needed to clarify long-term benefits.

E. Leadless pacing in patients with left ventricular assist devices

LP implantation has been reported in patients supported with left ventricular assist device (LVAD) who had prohibitive risk for transvenous pacemaker device implantation, particularly because of infection [92,93]. Although early experience suggests feasibility, the absence of large-scale or prospective studies limits definitive conclusions regarding safety and long-term outcomes in this population.

F. Leadless pacing in the very young and the very old

LPs are increasingly implanted across a broad age spectrum. In the pediatric population, LP implantation is feasible, with acceptable device parameters and complication rates similar to those observed in adult trials and registries [94,95]. Decisions regarding LP implantation in younger patients are typically individualized through shared decision-making, taking into account physical activity, quality of life, patient preferences, and cosmesis [94]. In older patients, LPs are often favored due to lower infection risk and limited vascular access for transvenous pacemaker implantation.

In younger adults, LP use is frequently driven by patient preference, given the absence of a subcutaneous pocket and improved cosmesis. However, LP implantation in young individuals may necessitate multiple procedures over a lifetime due to finite battery longevity (approximately 8–16 years), requiring device extraction and a new implant or the addition of new devices due to the previously implanted LP systems. In contrast, TVP systems typically require generator replacement alone, albeit with cumulative risks related to leads and pocket complications. The US-FDA has approved LP in very young individuals under ‘emergency use authorization’. The case series have reported successful LP implantation using an alternative internal jugular venous approach rather than femoral access [96,97].

G. Leadless pacing in congenital heart disease

Patients with congenital heart disease, both pediatric and adult, may develop sinus node dysfunction or high-grade atrio-ventricular block as part of the natural history of their condition or following surgical intervention [94]. TVP may be suboptimal in this population due to complex anatomy, congenital vascular anomalies, or sequelae of corrective or palliative surgery, as well as long-term risks associated with TVP leads and generator changes, including infections and vascular complications [98]. In pediatric patients, most TVP-related complications are lead-related, such as fractures caused by mechanical stress from an active lifestyle or trauma [98]. Long-term vascular complications, including venous stenosis, thrombosis, and occlusion, may also occur as leads traverse small, growing vessels. LPs eliminate transvenous leads and device pockets, thereby reducing these risks and offering potentially more durable pacing strategies in selected patients with congenital heart disease [98]. There are reported cases of LP implantation in a variety of adult congenital heart disease (ACHD) and following complex surgical repairs. These include patients with transposition of the great arteries (TGA) after a Senning procedure [99]; d-TGA following Mustard repair complicated by baffle stenosis [100]; Ebstein’s anomaly associated with atrial septal defect closure [101], and prior tricuspid valve surgery [102]; dextroposition of the heart; and repaired tetralogy of Fallot [103].

## 9. Extraction of LPs

Depending on pacing burden and device characteristics, the average lifespan of leadless pacemakers typically ranges between 8 and 16 years [104]. The decision to extract should balance procedural risk with the clinical need for removal. In most cases, particularly in the absence of infection or device malfunction, abandoning the device is preferred, as even a small RV can accommodate up to three Micra™ LPs. Transvenous extraction of leadless devices is a high-risk procedure that requires careful multidisciplinary planning and immediate surgical support. Generally, leadless pacemaker retrieval is reserved for specific indications, including pacemaker-related infective endocarditis, a significant rise in pacing threshold, a change in pacing indication to dual-chamber or CRT pacing, or device dislodgement [105,106].

Differences in fixation mechanisms, implant site, and the degree of device encapsulation, all of which can impact the technical difficulty and success of extraction [106]. The most frequently reported complications associated with the LP extraction include vascular injury (bleeding and hematoma), pericardial effusion or tamponade, device dislodgement or embolization, and valvular damage [107].

Currently available LPs differ significantly in their fixation design and retrieval options. Micra™ and Empower™ devices use a passive tine-based fixation system, while Nanostim™ and Aveir™ devices rely on an active screw-in helix fixation. Aveir™ pacemakers are specifically designed to be retrievable, even after long-term implantation, using a dedicated retrieval catheter that attaches to a docking button on the device. In contrast, Micra™ pacemakers are not designed for routine extraction after chronic implantation. When removal is required, it is usually performed with a snare technique through a femoral venous approach. Notably, successful retrieval of the Empower™ device using a dedicated retrieval catheter has been reported in only two cases to date [105,106,108].

As dual-chamber leadless pacemakers become more widely used and given the limited space in the right atrial appendage, atrial LP extractions may become increasingly necessary in the future to enable re-implantation at the end of life. Retrieval techniques are similar to those used for ventricular LPs, and a recent ovine study showed that atrial LP removal after 2 years of implantation is both safe and effective [109].

## 10. Future Direction

Replacing the LP at the end of battery life carries a nontrivial risk of procedural complications. To address the limitation of finite battery longevity, next-generation leadless technologies are actively exploring self-powered and self-rechargeable systems. Current preclinical in vivo studies are investigating methods to harvest kinetic energy from cardiac motion, convert it to electrical energy, and use it to support cardiac pacing [110,111,112]. Electromagnetic systems and piezoelectric materials have been utilized to generate electrical energy from ventricular wall motion [110,111,112]. If successfully translated into clinical practice, self-rechargeable LPs could fundamentally overcome one of the major constraints of current-generation devices.

Another important frontier in leadless pacing is the physiologic engagement of the conduction system. A recent first-in-human feasibility study demonstrated that leadless pacing systems can successfully engage the left bundle branch area, with promising early results [28]. Given that conventional right ventricular pacing is non-physiological and associated with a higher risk of PICM at high pacing burden, LBBAP has increasingly replaced traditional right ventricular pacing. It is conceivable that LBBAP with a traditional transvenous lead may eventually be supplanted by leadless approaches. However, larger studies with longer follow-up are required before widespread clinical adoption.

Utilization of LPs is also increasing among younger patients, driven by improved cosmesis, elimination of lead- and pocket-related complications, and avoidance of long-term vascular injury associated with transvenous leads [94]. Although there is concern about multiple LP implants over a lifetime due to battery longevity, newer-generation devices now offer improved durability. As device longevity, retrievability, and physiologic pacing capabilities continue to improve, the adoption of LPs among the younger patient population is likely to increase further. To overcome the need for vascular access with an intracardiac LP device and the finite battery lifespan that necessitates EOS-related complexities, the percutaneous pericardial pacing system is under active investigation [113].

## 11. Challenges in LP

Leadless pacemakers have finite battery lives, so limited longevity is a key challenge [30]. Unlike transvenous systems, where the generator can be replaced, an LP at EOS must be either abandoned in situ or percutaneously removed [114]. Each approach poses difficulties: multiple retained devices may increase intracardiac hardware burden, whereas extraction is technically challenging as fibrotic encapsulation develops over time. Dedicated retrieval tools and newer LP models aim to facilitate removal, but real-world extraction experience remains limited. Removal attempts carry a risk of complications (e.g., cardiac injury or device embolization), and current guidelines offer no specific recommendations for LP extraction. Case reports show that retrieval is feasible even after tissue ingrowth, but outcomes data are sparse, with the need for more evidence on EOS management [115]. At this time, to overcome this limitation, the risk of EOS management either abandoning or extracting the intracardiac leadless device, percutaneous leadless pericardial micro-pacemaker system implantation is under development. It has recently been reported in an animal study [113]. This technique lacks large bore vascular access and the cardiac and vascular complications associated with current-generation LP deployment; however, its safety and efficacy in humans have yet to be explored.

The LP implantation technique is still evolving, particularly LP engaging CSP, which is still under investigation. Some of the complications, their mechanisms, and ways to mitigate them are subjects of further research. Position of LP implantation, high septal versus apical septal, has been investigated in some observational studies and found to have implications for PICM and tricuspid regurgitation [34,35,36,37]. Likewise, some studies have shown similar or lesser rates of tricuspid regurgitation with LP compared with traditional transvenous pacemaker implantation [116]. In a study of individuals from Micra™ clinical trials who were considered precluded from traditional transvenous pacemaker implantation and received LP with Micra™, those who received LP had higher all-cause mortality, given higher rates of chronic comorbidities, with relatively low complication rates, and did not differ by preclusion status [117]. Future studies will elucidate the ideal position for LP implantation, procedural planning, and techniques to avoid potential complications.

Additionally, leadless pacing faces economic and system-level hurdles. The upfront cost of LP devices and implantation is significantly higher than for conventional pacemakers. For example, one analysis found that average total costs for LP implantation were more than double those for transvenous pacing (~€10,773 vs. ~€4572) [118]. However, reduced complication rates may render LP therapy cost-effective over time, and modeling studies have shown acceptable incremental cost-effectiveness ratios for LP despite higher initial costs. Beyond cost, barriers to broader LP adoption include limited operator experience and training, as well as the historical lack of dual-chamber pacing capability (addressed only recently by VDD/DDD leadless systems) [119]. These factors, combined with the absence of dedicated guideline recommendations, have tempered the integration of leadless pacing into clinical practice. However, with revolutionary developments and improvements in available tools and techniques, LP is increasingly integrated into modern electrophysiology as a day-to-day pacing modality.

## 12. Conclusions

Leadless pacing has progressed from a conceptual innovation to an established component of contemporary clinical practice. Given its lower rate of lead- and pocket-related complications and comparable outcomes to TVP, LP is increasingly favored in selected patient populations. As TVP has evolved with the adoption of conduction system pacing, leadless conduction system pacing represents a logical next milestone. The recent development of dual-chamber leadless pacing with reliable wireless communication and improved AV synchrony has further expanded the scope of clinical indications. In parallel, emerging modular leadless CRT platforms, combined with extra-vascular and subcutaneous ICDs, suggest a future in which fully leadless pacing-defibrillation strategies may become feasible in routine practice. Collectively, these innovations indicate that leadless technologies have the potential to fundamentally reshape cardiac rhythm management in the years ahead.

## Figures and Tables

**Table 1:** Major timeline of leadless pacing development.

Year	Author	Leadless Pacing Development
1970	Spickler et al. [3]	Self-contained leadless cardiac pacing in a canine model
2012	Reddy et al. [4]	First-in-human LP (Nanostim™ LP)
2022	Knops et al. [27]	First dual-chamber LP (Aveir™ DR)
2024	Reddy et al. [28]	First-in-human leadless pacemaker system for left bundle branch area pacing (LBBAP)
2025	Hollis et al. [29]	First-in-human Bachman bundle pacing

**Table 2:** Currently available LPs in clinical practice/studies (Aveir™; Micra™; Empower™).

FEATURES	AVEIR™ VR ^ϕ^	AVEIR™ AR ^ϕ^	MICRA™ VR2 ^ψ^	MICRA™ AV2 ^ψ^	EMPOWER™ ^γ^
	^ 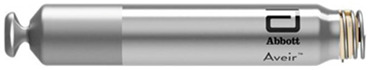 ^	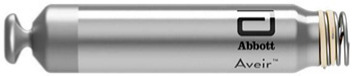	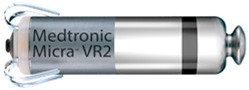	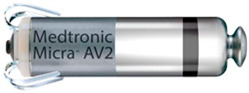	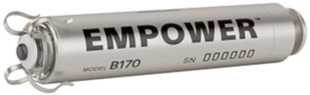
**Dimensions (mm)**	38 × 6.5	32.2 × 6.5	25.9 × 6.7	25.9 × 6.7	32.1 × 6.0
**Volume (cc)**	1.1	1.0	0.8	0.8	0.75
**Pacing mode**	VVI(R)	AAI(R)	VVI(R)	VVI(R) or VDD(R)	VVI(R) + S-ICD–directed ATP
**Dual-chamber LP capable**	Yes	Yes	No	No	No
**Fixation mechanism**	Screw-in helix	Dual screw-in helix	Four nitinol tines	Four nitinol tines	Four nitinol tines
**Battery type**	Lithium carbon monofluoride	Lithium carbon monofluoride	Lithium-hybrid carbon monofluoride/silver vanadium oxide	Lithium-hybrid carbon monofluoride/silver vanadium oxide	Lithium carbon monofluoride
Battery longevity (years)ISO setting *	9.9 (VVIR) 7.3 (DDDR)	6.8 (VVIR) 5.0 (DDDR)	4.7 (VVIR)	4.8 (VDD)	N/A
**Battery longevity (years) Alternative setting †**	16.1 (single-chamber) 6.8 (dual-chamber)	11.2 (single-chamber) 6.8 (dual-chamber)	12.4	11.6	N/A
**Rate-responsive sensor**	Temperature	Temperature	3-axis accelerometer	3-axis accelerometer	—
**MRI compatibility**	1.5 T, 3 T	1.5 T, 3 T	1.5 T, 3 T	1.5 T, 3 T	1.5 T, 3 T
**Remote monitoring**	No	No	CareLink™	CareLink™	No
**Magnet response**	VOO 100 bpm × 8 cycles, then battery-dependent rate	AOO (VOO with dual-chamber pacing) 100 bpm × 5 cycles, then battery-dependent rate	No	No	N/A
**Dedicated retrieval catheter**	Yes	Yes	No	No	No

* ISO standard setting: 2.5 V at 0.4 ms, 60 bpm, 100% ventricular pacing, 600 Ω, † Alternative setting: As specified per manufacturer (lower voltage, shorter pulse width, 500 Ω load). ^ϕ^ Image provided courtesy of Abbott. AVEIR, Abbott, Abbott ‘A’ device are trademarks of Abbott or its related companies. Reproduced with permission of Abbott. © 2025 Abbott. All rights reserved. ^ψ^ Image provided courtesy of Medtronic. ©2025 Medtronic. All rights reserved. Used with the permission of Medtronic. ^γ^ Image provided courtesy of Boston Scientific. ©2026 Boston Scientific Corporation or its affiliates. All rights reserved.

**Table 4:** Summary of leadless pacing trials with AV-synchronization (AVS) at rest and with activity.

Landmark Studies	Study Design	Sample Size N	Primary Endpoint	Secondary Endpoint	LP Model	Follow-Up Duration (mo)	AVS at Rest	AVS at the Activity
**MARVEL** [25]	Prospective nonrandomized multicenter study	70	Rate of AVS during the 30-min resting period	Rate of atrial detection (A4)	Micra™ AV	6	87% (range 30.2–100)	62.7–84.0%
**MARVEL-2** [26,53]	Prospective, multicenter, nonrandomized clinical trial	77	Primary efficacy: ≥70% P-waves with ventricular capture ≤300 ms (VVI & VDD) Primary safety: No pauses > 2 cycles; no oversensing tachycardia > 100 bpm > 3 min	LVOT-VTI (VVI vs. VDD)	Micra™ AV	9.7	89.2% (CI: 84.8–92.5)	69.8–86.7%
**AccelAV** [54]	Prospective, nonrandomized, multicenter, single-arm clinical trial	152	Resting AV synchrony (%) at 1 month in complete AV block + normal sinus rhythm	AVS stability at 3 months; 24-h ambulatory AVS; LVOT-VTI (stroke volume); QoL (EQ-5D-3L)	Micra™ AV	3	84.1% (CI 78.3–88.6)	74.5% (CI 70.4–78.2)
**Aveir DR i2i Study** [27]	Prospective multicenter single-group	300	90-day safety; atrial capture/sensing + ≥70% AV synchrony at 3 months	Secondary safety & performance endpoints	Aveir™ DR	3	98.0% (CI 97.0–99.1)	96.5–98.4%

**Table 5:** Main studies on the WiSE-CRT system.

Studies	Study Design	Sample Size	Primary Endpoint	Secondary Endpoint	Follow-Up (mo)	Procedural Success n(%)	Short-Term Complications n(%)	Improved HF Symptoms (%)	Echo Response at 6 Months
**WiSE-CRT study** [67]	Prospective multicenter feasibility study	17	Biventricular pacing capture on ECG at 1 month + device/procedure safety	Biventricular pacing performance and clinical/echo response at 6 months	6	13 (76.5)	6 (35) procedural complications	8 (66.7) ≥ 1 NYHA class	-
**SELECT-LV** [70]	Prospective multicenter non-randomized feasibility trial	35	Biventricular pacing on ECG at 1 month + device/procedure safety (≤30 days)	Clinical composite and echocardiographic CRT response at 6 months	6	34 (97.1)	3 (8.6) procedural; 8 (22.3) intermediate complications ^c^	28 (84.8)	21 (66) ^a^
**SOLVE-CRT roll-in phase I study** [68]	Prospective multicenter nonrandomized roll-in phase	31	Primary safety: Type-I device/procedure-related complications through 6 mo	Primary efficacy: Mean % change in LVESV at 6 mo	6	31 (100)	3 (9.7) type-I and 5 (16.1) non-type-I complications	14 (46.7) ≥ 1 NYHA class	12(41.4) ^a^; 10 (34.5) ^b^
**SOLVE-CRT trial** [71]	Prospective multicenter trial (randomized + single-arm)	183 (all 3 phases)	Safety: Freedom from Type-I device/procedure complications at 6 mo; Efficacy: Mean % reduction in LVESV at 6 mo	Biventricular pacing %, APCT stability, LVEF & KCCQ response	6	99/108 (91.7) in part 2; 75 (100) in part 3	35 (19.1) type-I ^d^	49 (55.7) ≥ 1 NYHA class	41 (46.1) ^a^
**WiSE-CRT PMR** [72]	Prospective multicenter international registry (real-world post-market)	90	Procedural success and safety (acute, intermediate, chronic complications)	Clinical response at 6 mo	6	85 (94.4)	4 (4.4) procedural; 17 (18.8) intermediate complications ^c^	60 (69.8)	25 (58.1) ^b^

^a^ Increase in EF > 5%; ^b^ 15% reduction in LVESV; ^c^ 24 h to 1 month; ^d^ at 6 months.

## Data Availability

There is no new data were created.

## Supplementary Materials

The following supporting information can be downloaded at: https://www.mdpi.com/article/10.3390/jcm15031251/s1, Table S1. Summary table outlining the principal clinical trials on leadless pacing, including study design, primary and secondary endpoints, main findings, and duration of follow-up.

## Abbreviations

The following abbreviations are used in this manuscript:

ATP	anti-tachycardia pacing
AV	atrioventricular
BB	Bachmann’s bundle
BBAP	Bachmann’s bundle area pacing
CE	Conformité Européenne
CRT	cardiac resynchronization therapy
CSP	Conduction system pacing
EV-ICD	extravascular implantable cardioverter-defibrillator
GDMT	guideline-directed medical therapy
i2i	implant-to-implant
LBBB	left bundle branch block
LBBAP	left bundle branch area pacing
LP	leadless pacemaker
NYHA	New York Heart Association
PICM	pacing-induced cardiomyopathy
PMS	pacemaker syndrome
S-ICD	subcutaneous implantable cardiac defibrillator
TVP	transvenous pacing
TR	Tricuspid regurgitation
US-FDA	United States Food and Drug Administration
WiSE-CRT	Wireless Stimulation Endocardially for Cardiac Resynchronization
