# Supplementary material for: Era of Synchronized Physiologic Leadless Pacing: A Novel Approach to Cardiac Pacing and Ongoing Development
Source: J Clin Med. 2026 Feb 4;15(3):1251. doi: 10.3390/jcm15031251 (PMC12897699; doi:10.3390/jcm15031251)
Supplement: Supplementary file 1 [file jcm-15-01251-s001.zip › jcm-4093056-supplementary.pdf]

Supplementary materials

Table S1. Summary table outlining the principal clinical trials on leadless pacing, including study design, primary and secondary endpoints, main findings, and duration of follow-up

Landmark Studies	Study Design	Sample Size	Primary endpoint	Secondary endpoint	LP Model	Follow-up duration (mo)	Primary result	Secondary results
**LEADLESS study [4,21]**	Prospective, nonrandomized, single-arm multicenter feasibility study	33	Freedom from complications at 90 days	Implant success; pacing/sensing performance; rate-responsive function	Nanostim	38	Complication-free rate was 94% (31/33)	After 3 months, the pacing performance (sensing, impedance, and pacing threshold) were stable within the accepted range.
**LEADLESS-II [22]**	Prospective nonrandomized multicenter IDE trial	N=526 (primary cohort n=300)	Efficacy: acceptable pacing threshold ≤2.0 V & R-wave ≥5 mV at 6 mo;<br>Safety: freedom from device-related SAEs at 6 mo	All device- and non-device SAEs	Nanostim	6	Efficacy: 270/ 300 patients (90.0%; 95% CI, 86.0-93.2, P=0.007), and the safety: 280/300 patients (93.3%; 95% CI, 89.9-95.9; P<0.001).	Device-related serious adverse events were observed in 6.7%
**MICRA-IDE [25]**	Prospective, nonrandomized, single-group, multicenter international IDE trial	725	Safety: Freedom from system- or procedure-related major complications at 6 mo;<br>Efficacy: Low & stable pacing capture threshold ≤2.0 V at 6 mo	Comparison vs historical transvenous cohort	Micra-VR	16.4	Primary safety end point: 96.0% (95% CI, 93.9-97.3; P<0.001)<br>Primary efficacy end point: 98.3% (95% CI, 96.1-99.5; P<0.001)	28 major complications in 25 patients, significantly fewer major complications than control patients (HR, 0.49; 95% CI, 0.33-0.75; P=0.001)
**MICRA-CED [40]**	Nationwide observational comparative study	6219	3-year chronic complications	Reinterventions, HF hospitalization, all-cause mortality	Micra-VR	22.2	32% lower rate of chronic complications compared with transvenous patients ([HR] 0.68; 95% CI, 0.59–0.78)	41% lower rate of reintervention compared with transvenous patients (HR 0.59; 95% CI 0.44–0.78)

**MICRA-PAR [41]**	Prospective, non-randomized, multicenter post-approval registry	1809	System- or procedure-related major complications through 60 months	System revisions; mortality	Micra-VR	51.1	4.5% [95% CI: 3.6%-5.5%]; (HR: .47, 95% CI: .36-.61; P < .001)	all-cause system revision rate at 60 months: 4.9% (95% CI: 3.9%-6.1%); all-cause mortality rate of 39.5%
**MICRA AV-CED [42]**	Medicare claims–linked, observational comparative cohort (Micra-AV vs dual-chamber TVP)	7471	30-day acute complication rate	6-month chronic complications, reinterventions, mortality	Micra-AV	6	Unadjusted rate: (9.1% vs 8.7%; P = .61), Adjusted rate: (8.6% vs 11.0%; P < .0001).	Rates of complications (adjusted HR 0.50; 95% CI 0.43–0.57; P < .0001) and reinterventions (adjusted HR 0.46; 95% CI 0.36–0.58; P < .0001).
**LEADLESS-II phase II [43]**	Prospective, multicenter, single-arm pivotal IDE trial	210	Freedom from serious adverse device effects at 1 yr	Electrical performance success (pacing threshold ≤2.0 V & R-wave ≥5 mV at 1 year)	Aveir-VR	14.4	Freedom from serious adverse device effects: 93.2% (95% CI: 88.7%-95.9%)	Effectiveness criteria met in: 195 (95.1%) (95% CI: 91.2%-97.6%)
**Aveir DR i2i Study [28]**	Prospective, multicenter, single-group first-in-human trial	300	Freedom from device- or procedure-related serious adverse events at 90 days	Atrial capture + sensing performance and ≥70% AV synchrony at 3 months	Aveir-DR	3	271 patients (90.3%; 95% CI, 87.0-93.7)	Atrial capture threshold and sensing amplitude: 90.2% (95% CI, 86.8-93.6) ≥70% AV synchrony: 97.3% of the patients (95% CI, 95.4- 99.3)
